# Development and Validation of an Anthropometric Equation to Predict Fat Mass Percentage in Professional and Semi-Professional Male Futsal Players

**DOI:** 10.3390/nu14214514

**Published:** 2022-10-27

**Authors:** Rita Giro, Catarina N. Matias, Francesco Campa, Diana A. Santos, Margarida L. Cavaca, Pedro Duque, Marco Oliveira, Nuno Matos, Filipa Vicente, Paula Pereira, Heitor O. Santos, Grant M. Tinsley, Filipe J. Teixeira

**Affiliations:** 1 Bettery LifeLab, Bettery S.A., Porto Salvo, 2740-262 Lisboa, Portugal; 2 Research Center on Sports, Physical Education, Exercise and Health (CIDEFES), Universidade Lusófona, 1749-024 Lisboa, Portugal; 3 Department of Biomedical Sciences, University of Padua, 35131 Padua, Italy; 4 Interdisciplinary Center for the Study of Human Performance (CIPER), Faculdade de Motricidade Humana, Universidade de Lisboa, Cruz-Quebrada, 1499-002 Lisboa, Portugal; 5 School of Education and Social Sciences (ESECS), Polytechnic Institute of Leiria, 2411-901 Leiria, Portugal; 6 Grupo de Estudos em Nutrição Aplicada (GENA), CiiEM, Egas Moniz-Cooperativa de Ensino Superior, Almada, 2829-511 Setúbal, Portugal; 7 Clube Recreativo Leões de Porto Salvo, Porto Salvo, 2740-025 Lisboa, Portugal; 8 School of Medicine, Federal University of Uberlandia (UFU), Uberlandia 38408-100, Minas Gerais, Brazil; 9 Department of Kinesiology & Sport Management, Texas Tech University, Lubbock, TX 79409, USA; 10 Atlântica—Instituto Universitário, Fábrica da Pólvora de Barcarena, Barcarena, 2730-036 Lisboa, Portugal

**Keywords:** athletic performance, body composition, futsal, kinanthropometry, nutrition, team sport

## Abstract

This study aimed to (i) characterise the body composition of professional and semi-professional male futsal players, (ii) assess the validity of commonly used equations to estimate FM%, (iii) develop and cross-validate a futsal-specific FM% prediction equation. In a cross-sectional design, 78 adult male futsal players were assessed for body mass, stature, skinfolds, and girths as per the International Society for the Advancement of Kinanthropometry protocol and completed a dual-energy X-ray absorptiometry (DXA) scan for reference body composition data. Using paired-sample *t*-tests, the FM% from the DXA and nine published equations were compared. New sport-specific models were developed by stepwise multiple regression. Existing equations were cross-validated using the least squares regression, concordance correlation coefficient, and the Bland–Altman analyses. New equations were further cross-validated using the PRESS approach. None of the existing equations accurately predicted the DXA-derived FM% (*p* < 0.001; R^2^ ≤ 0.76, SEE ≥ 1.59; CCC ≤ 0.83; bias = −8.2% to −1.3%, limited agreement, and varying trends). The novel Bettery^®^ equation: −0.620 + (0.159 ∗ Σ4SKF [triceps, abdominal, iliac crest, and front thigh (mm)]) + (0.120 ∗ waist girth (cm)), demonstrated a high accuracy (R^2^ = 0.85, SEE = 1.32%), a moderate strength of agreement (CCC = 0.92), no bias (0.2%), good agreement (±2.5%), and no trend (*r* = −0.157; *p* = 0.170) against the DXA. The Bettery^®^ equation is the first to allow for a valid and sport-specific assessment of FM% in male futsal players.

## 1. Introduction

Futsal is the five-a-side version of football (soccer) endorsed by the Fédération Internationale de Football Association (FIFA). Merging the rules and regulations of several team sports, futsal is played on an indoor court during a 2 × 20-min-game with unlimited substitutions, which can last 75–85% longer due to pauses in time with each dead ball. Therefore, futsal is characterised by a greater number of high-intensity actions than football and other intermittent sports [1], and futsal players must display great sprinting abilities, leg muscle power, and technical skills in order to be successful [2,3], despite relying predominantly on the aerobic energy pathways for fuel [4]. To accomplish increased power and strength during exercise, higher muscle-to-fat ratios are necessary [3]. Thus, it is common practice for technical teams to monitor athletes’ body compositions over time, to further provide information regarding nutritional and training strategies aiming to improve performance [5,6]. Indeed, a greater fat mass (FM) in futsal players has been associated with a reduced aerobic capacity [7], a lower vertical jump height, and increased fatigue [8].

In athletes, the most suitable technique to assess body composition should be chosen considering the relevant context [6]. In a laboratory setting, dual-energy X-ray absorptiometry (DXA) is often deemed a ‘criterion standard’ to assess body composition in the scope of sport [9]. However, this methodology is technically complex, expensive, and inaccessible to most sports professionals, which makes the implementation of frequent body composition evaluations difficult. In field settings, surface anthropometry (SA) is the most popular method to assess changes in athletes’ body composition [10] as it is practical, low-cost, and minimally affected by biological variability [11]. Despite their limitations relating to accuracy, anthropometric measurements have proven to generate reliable data when procedures are conducted according to the standards of the International Society for the Advancement of Kinanthropometry (ISAK), by an accredited anthropometrist, and using high-quality, well-calibrated instruments [6]. Although the use of raw data (i.e., sums of skinfolds) is encouraged when tracking FM changes, the fact that practitioners may monitor different sums of skinfolds (e.g., three, four, seven, and eight skinfolds) and the lack of normative data in various sports populations to interpret the values obtained, complicates the recommended shift from the broad utilisation of FM percentage (FM%) to the sum of the eight ISAK restricted profile skinfolds [9]. Under such circumstances, the preference for a validated, sport-specific prediction equation may offer improved accuracy in the estimation of FM% [5,12].

To date, few studies have assessed the body composition profile of male futsal players at the professional and semi-professional level, and no anthropometric equations have been developed or validated in this specific population. Thus, the main goals of the present study were to characterise the body composition of adult male futsal players, assess the validity of commonly used anthropometric equations to predict FM%, and to develop and validate a sport-specific anthropometric equation to accurately estimate FM% in male futsal players.

## 2. Materials and Methods

### 2.1. Participants

The sample size was calculated according to the PRESS approach, using the FM% as a primary outcome, and considering a medium-to-small effect size with a type I error of 5% and a power of 95%. A total of 78 futsal players were included in the study, 54 of which were professional players competing in the Major Portuguese Futsal League “LIGA PLACARD”, including players on the Portuguese National Team. The training load of the professional players was 5 sessions of 150 min and 1 official game per week. The remaining 24 players were semi-professional athletes competing in the 2nd and 3rd National Futsal Leagues, with 3 training sessions of 150 min and 1 official game per week. All volunteers proved eligible by presenting ≥18 years old, playing futsal at national or international level, and not taking any medication or supplementation known to interfere with body composition. The data collection was conducted at the Bettery^®^ LifeLab, Lisbon, Portugal during the competitive season, between November 2021 and March 2022. A written informed consent was obtained from all participants and ethical approval was provided by the Faculty of Human Kinetics Institutional Review Board (approval number 37/2021), attesting to the fulfilment of all human research standards set out by the declaration of Helsinki [13]. This work stems from the 4BETTPRO project, registered at clinicaltrials.gov (NCT05228236). More information on the study design may be obtained from our previous work [14].

### 2.2. Study Design

In a cross-sectional design, the athletes’ body compositions were assessed to validate existing FM% anthropometric equations, and to develop and cross-validate new, futsal-specific FM% prediction equations against a reference method (DXA). The participants were instructed to refrain from vigorous exercise and the consumption of alcohol and caffeine or other stimulants in the 12 h preceding the evaluation. Additionally, they were instructed to maintain their usual diet on the day before the test and no subjects were on creatine supplementation. Assessments were performed successively on the same morning, in an overnight-fasted state (i.e., abstention from food and fluids overnight), and on an empty bladder.

#### 2.2.1. Dual-Energy X-ray Absorptiometry

The participants underwent a whole-body DXA scan (Horizon Wi, Hologic, Waltham, MA, USA) according to the procedures recommended by the manufacturer. The same technician positioned the patient, performed the scan, and executed the analyses, in a ventilated room with controlled temperature and humidity. The DXA measurements of the whole-body bone mineral content (BMC, kg), fat-free mass (FFM, kg), lean soft tissue (LST, kg) and visceral adipose tissue (VAT, cm^2^), as well as absolute mass (kg) and FM% were analysed. The test-retest technical error of measurement (TEM) for the FM% in 29 participants was 1.7%.

#### 2.2.2. Surface Anthropometry

The participants had their body mass and height measured to the nearest 0.1 kg and 0.1 cm, respectively, using a scale and a wall stadiometer (Seca, Hamburg, Germany). The body mass index (BMI) was calculated as body mass (kg) divided by squared stature (m^2^). The eight skinfold thicknesses (triceps, subscapular, biceps, iliac crest, supraspinale, abdominal, front thigh, and medial calf) and five girths (arm relaxed, arm flexed and tensed, waist, gluteal, and calf) from the ISAK restricted profile were measured with an accuracy of 0.1 mm and 0.1 cm, respectively, using a Harpenden skinfold calliper (Baty International, Burgess Hill, England) and an anthropometric measuring tape (CESCORF, Porto Alegre, Brazil). Two measurements were taken per site and a third one was obtained when the TEM was >5%. Either the mean of the two measurements, or the median of the three measurements, was considered for analysis. All anthropometric measurements were performed by a level I-accredited anthropometrist according to the standards of the ISAK [15]. The participants wore minimal clothing and no shoes during the assessment, conducted in a private environment. The anthropometrist’s test-retest TEM for the measurement of the same skinfolds and girths in 29 participants ranged between 0.10–2.24%.

#### 2.2.3. Validity of Published Anthropometric Equations

The FM% was estimated from nine anthropometric equations (Supplementary Table S1) selected from recent overviews of FM% prediction equations for male football players [16,17] or newer equations developed in team sport athletes [18,19]. Athlete-specific and generalised equations were considered when (1) adult males were included in the regression analysis, and (2) the equations only included ISAK restricted profile sites and had been developed using a Harpenden skinfold calliper [15]. The results from body density prediction equations were converted to FM% using Siri’s formula [20].

### 2.3. Statistical Analysis

IBM SPSS Statistics v.28.0.1.0, 2021 (IBM, Chicago, IL, USA) was used for preliminary data analysis. All variables were assessed for normality (Kolmogorov–Smirnov, *p* > 0.05) and described (mean ± standard deviation for the normally distributed variables, or median (25th–75th percentiles) for age, the only non-normally distributed variable in this study). The Mann–Whitney *U* and independent sample *t*-tests were used to compare professional and semi-professional players on age or other variables, respectively. Ninety-five percent confidence intervals (95% CI) were reported for the differences. Preliminary Pearson correlations were analysed to identify associations between the skinfold thicknesses and the DXA-derived FM%. The skinfolds from each body segment (upper limb, trunk, and lower limb) with the strongest Pearson correlation [21] were selected to calculate the sums of 3, 4, and 8 skinfolds. To develop sport-specific equations, stepwise multiple regression analyses were carried out exploring the ability of the sums of skinfolds, individual girths, body mass, stature, ethnicity, and/or age to predict FM%. The significance for inclusion was set at *p* ≤ 0.05 and for removal at *p* ≤ 0.1. Normality and homoscedasticity of the residuals in the new models were confirmed. Multicollinearity was tested between the predictor variables in each model using the variance inflation factor ≤10 as the criterion [22].

The cross-validation of existing equations was three-fold. Firstly, paired sample *t*-tests were conducted to check for differences between the DXA-derived FM% and the equation-predicted FM%, and an ordinary least squares regression was performed. Subsequently, the concordance correlation coefficient (CCC) between the methods was calculated on MedCalc Statistical Software v.11.1.1.0, 2009 (Mariakerke, Belgium) as per Lin’s methodology [23] including a measure of precision (ρ) and accuracy (C_b_). Lastly, the Bland–Altman analysis [24] was conducted on GraphPad Prism 9 (Graph Pad Software, Inc., San Diego, CA, USA) to assess the fixed bias (mean difference), 95% limits of agreement (LoA), and proportional bias (trends) in FM% estimation, by plotting the differences against the means of the methods. In the newly developed equations, the prediction residual error sum of squares (PRESS) statistic [25] was additionally performed using SigmaPlot v.11.0 (Systat Software, Inc., Düsseldorf, Germany), as previously described by our group [26], in order to limit overfitting and selection bias. The PRESS is an internal cross-validation ‘leave-one-out’ approach where an unbiased coefficient of determination (R^2^) and standard error of the estimate (SEE) are derived to better assess model predictive capability. Statistical significance was set at *p* < 0.05.

## 3. Results

No significant differences were found between athletes of different competition levels for the DXA variables (all *p* > 0.05). Only age (26 [22–32] vs. 20 [19–24] yrs; 95% CI = 2 to 7; *p* < 0.001), BMI (23.9 ± 0.3 vs. 22.6 ± 0.4 kg/m^2^; 95% CI = 0.3 to 2.2; *p* = 0.009), arm-girth relaxed (30.4 ± 0.2 vs. 29.0 ± 0.4 cm; 95% CI = 0.5 to 2.3; *p* = 0.003), and arm-girth flexed and tensed (32.9 ± 0.3 vs. 31.4 ± 0.3 cm; 95% CI = 0.6 to 2.4; *p* = 0.001) were higher in professional compared to semi-professional players, while stature was lower (174.4 ± 0.9 vs. 179.4 ± 1.3 cm; 95% CI = −8.2 to −1.7, *p* = 0.003). As none of the variables of interest showed differences between the methodologies, descriptive statistics are presented for the whole sample (Table 1).

Table 2 displays the cross-validation results for each published equation evaluated in this study. All the equations predicted FM% differently from the DXA (all *p* < 0.001) with 60–76% explanatory power, SEE values between 1.59–2.05%, and poor strength of agreement (CCC ≤ 0.83). The Bland–Altman analyses revealed that all the equations systematically underestimated the DXA-derived FM% (bias = −8.2% to −1.3%) with wide 95% LoA (−13.0% to 2.6%), except for the Suarez-Arrones which slightly overestimated it (bias = 1.0%; 95% LoA = −2.4 to 4.5%). The Lohman, Reilly, and Zemski Caucasian demonstrated very high (*r* = −0.907), modest (*r* = −0.665), and low (*r* = −0.278) tendencies, respectively, to produce larger underestimations at higher FM%s. On the other hand, Stewart (*r* = 0.569), Withers, Durnin–W, and Durnin–R (*r* ≤ 0.294; *p* < 0.05) displayed modest and low trends, respectively, towards underestimation at a lower FM%. The Suarez-Arrones and Evans 3SKF presented a fixed bias across the whole FM% range (*p* < 0.05). Overall, the Suarez-Arrones demonstrated the best results, but none of the equations accurately predicted FM% in our sample. Hence, new prediction models were developed.

Table 3 shows the preliminary identification of the skinfolds that were most associated with the DXA-derived FM% per body segment.

The sum of the eight skinfolds (Σ8SKF, all measured) was highly positively correlated to the DXA-derived FM% (*r* = 0.878), similarly to the sum of three skinfolds (Σ3SKF): triceps, abdominal, and front thigh (*r* = 0.881), while the sum of four skinfolds (Σ4SKF): triceps, iliac crest, abdominal, and front thigh produced a very high positive correlation to the DXA-derived %FM (*r* = 0.909) (all *p* < 0.001). Despite these interesting results, not only the Σ4SKF and Σ3SKF, but also the Σ8SKF were tested by stepwise multiple regression, aiming to clarify the superiority of the FM% prediction models using the sums of fewer skinfolds.

The initial population-specific FM% prediction models developed by multiple linear regression, and cross-validated using the PRESS statistic are presented in the Supplementary Tables S2 and S3. Table 4 displays results for the best three equations, only.

Upon application of the backward stepwise procedure, all the variables, but the Σ3SKF, Σ4SKF, Σ8SKF, waist girth and/or age, were removed, due to not improving the fit of the model. The prediction models including the Σ3SKF, Σ4SKF, or Σ8SKF were most improved by the addition of the waist girth (R^2^ ≥ 0.80; SEE ≤ 1.52%; Table 4). However, age was not a significant predictor of the DXA-derived FM% in any of the models where it was included (*p* > 0.05; Table S2). The PRESS cross-validation revealed no substantial changes in accuracy. The models performed equally well when the sum of the skinfolds was coupled with waist girth only, or with waist girth and age (PRESS R^2^ ≥ 0.80; PRESS SEE ≤ 1.80%; Table S3). The PRESS R^2^ and SEE were worse in the models including the sum of the skinfolds only, or the sum of the skinfolds and age. Considering the statistical performance and utility of the models, those including the sum of the skinfolds and waist girth were submitted to further testing.

**Figure 1:**
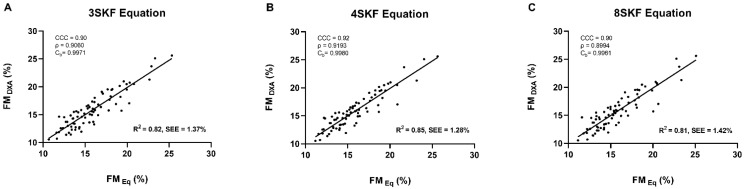
The least squares regression (R^2^, coefficient of determination; SEE, standard error of the estimate) and concordance coefficient of correlation (CCC), including the precision (ρ) and accuracy (C_b_) analyses of the fat mass percentage from the reference method (FM_DXA_ (%)) vs. the novel prediction equations (FM_Eq_ (%)) in high-level male futsal players (*n* = 78). The plots indicate the line of regression. The prediction equations are (**A**) the sum of three skinfolds (triceps, abdominal, and front thigh), waist girth; (**B**) the sum of four skinfolds (triceps, iliac crest, abdominal, and front thigh), waist girth; (**C**) the sum of eight skinfolds (triceps, subscapular, biceps, iliac crest, supraspinale, abdominal, front thigh, and medial calf), waist girth. No significant differences between the reference and predicted fat mass percentage by any of the equations (all *p* > 0.05).

Figure 1 represents the least squares regression and CCC analyses between the DXA-derived FM% and the predicted FM% by the Σ3SKF (A), Σ4SKF (B), and Σ8SKF (C) with the waist girth equations. Based on the least squares regression, the Σ4SKF equation produced the single best prediction of the reference FM%, accounting for 85% of the variance in the DXA-derived FM%. The three prediction equations showed a moderate strength of agreement (CCC = 0.90–0.92) with the DXA.

**Figure 2:**
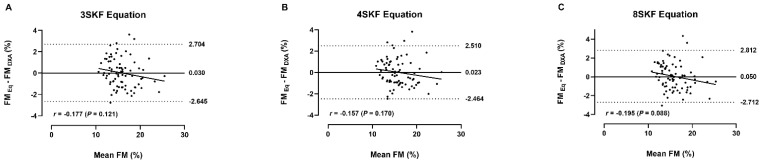
The Bland–Altman plots between the fat mass percentage differences and means of the reference method (FM_DXA_ (%)) and the novel prediction equations (FM_Eq_ (%)) in high-level male futsal players (*n* = 78). The solid line represents the mean difference (fixed bias), the dotted lines indicate the 95% limits of agreement (±1.96 SD) and trend illustrates the correlation (*r*) between the difference of the methods and the mean of the methods (proportional bias). The prediction equations are (**A**) the sum of three skinfolds (triceps, abdominal, and front thigh), waist girth; (**B**) the sum of four skinfolds (triceps, iliac crest, abdominal, and front thigh), waist girth; (**C**) the sum of eight skinfolds (triceps, subscapular, biceps, iliac crest, supraspinale, abdominal, front thigh, and medial calf), waist girth. No significant correlations between the difference and the mean of the methods (all *p* > 0.05).

Figure 2 shows the Bland–Altman plots between the DXA-derived FM% and the predicted FM% by the Σ3SKF (A), Σ4SKF (B), and Σ8SKF (C) equations. The individual variability in FM% ranged from −2.7 to 2.8% (95% LoA) and no trends were observed between the differences and means of the methods (all *p* > 0.05).

Bearing all of this in mind, the final anthropometric equation selected for FM% estimation due to exhibiting the best overall performance (Bettery^®^ Equation) was:

%FM = −0.620 + 0.159 ∗ Σ4SKF (mm) + 0.120 ∗ waist girth (cm)

where Σ4SKF = the sum of the triceps, abdominal, iliac crest, and front thigh skinfolds.

## 4. Discussion

The present study is the first to develop and validate a futsal-specific anthropometric equation to estimate the FM% in adult male high-level players. We propose an equation using only the Σ4SKF and waist girth which explains 85% of the variance in the DXA-derived FM% with a low random error (SEE = 1.32%) and a moderate strength of agreement (CCC = 0.92). Moreover, a low individual variability (95% LoA = ±2.5%) and no fixed or proportional bias were observed, indicating that the Bettery^®^ Equation for FM% estimation is valid at both the group and individual level, across the range of the FM%s observed in these athletes. Conversely, the commonly used equations to estimate FM% in athletes proved inaccurate in our sample. To the best of our knowledge, this is also the first study to characterise the Σ8SKF and girths recommended by ISAK in futsal players, which may support the development of normative body composition data for this sports population and prompt the utilisation of raw anthropometric measures to monitor body composition in the sport.

The professional and semi-professional players in our study did not differ insofar as the main outcome variables, namely body mass, Σ8SKF, and the DXA-derived FM% (*p* > 0.05). Data from south European futsal players with similar training loads are in line with our findings by demonstrating a mean body mass of about 72–75 kg and a FM of about 15% (11 kg), albeit assessed by various methodologies [1,3,33,34]. Interestingly, no evidence of comparable Σ8SKF was found. On the other hand, we observed differences between professional and semi-professional players regarding age, stature, BMI, and upper body SA (i.e., arm-girth relaxed, and arm-girth flexed and tensed).

Although several FM% prediction equations exist, none to date have included male futsal players in their development. This fact likely explains the limited validity of the athlete-specific and generalised anthropometric equations assessed in this study, despite the strict inclusion criteria that were established. Indeed, the best cross-validation results were found for the FM% (not body density) prediction equations developed in European, male football players, following ISAK standards and using the DXA as the criterion method (i.e., Suarez-Arrones > Reilly > Stewart; Table 2). Notwithstanding, all but one equation estimated the DXA-derived FM% with clear underestimation bias (−8.2 to −1.3%) and considerable individual variability. Considering that professional male futsal players have been shown to present a higher FM% than footballers [8]; and that futsal forwards display a slightly higher FM% (16.5%) than basketball (14.5%) and handball (14.6%) players of the same position [34], these observations were expected. Futsal is a very demanding game, interchanging high-intensity anaerobic sprints and high-power movements with aerobic actions and dead ball moments. The greater proportion of high-intensity vs. endurance actions in futsal compared to other team sports [1] may explain this phenomenon. Supporting these findings are the recommendations that equations should be sport-specific for a more accurate FM% prediction in athletes [5]. Therefore, futsal-specific FM% prediction equations were developed in the present study.

As previously outlined in the literature, solely monitoring the upper body sites may not provide an accurate depiction of the whole-body composition in team sport athletes [5,35], since the lower limbs are highly trained and recruited during exercise. Thus, we considered the biological relevance of the anthropometric sites selected for the prediction model development, by integrating the upper (triceps), trunk (abdominal and iliac crest), and lower body (front thigh) skinfolds into the calculation of the Σ3SKF and Σ4SKF (Table 3), in line with the best-performing published equations examined in this study. It is noteworthy that the Σ4SKF was more highly correlated to the DXA-derived FM% than the Σ8SKF (*r* = 0.909 vs. *r* = 0.878; *p* < 0.001). From a practical perspective this is a great advantage, as it allows the practitioner to minimise the time spent in data collection and eases the burden on the athlete. However, monitoring the Σ8SKF is currently deemed as best practice in applied sport [9] and could provide a finer representation of the regional and individual changes in FM over time than the sums of fewer skinfolds [36]. Therefore, the best predictor combinations of the DXA-derived FM% using the Σ3SKF, Σ4SKF, or Σ8SKF (i.e., with waist girth; Table 4) were validated in this study, hoping to serve the needs of all sports professionals, from the most practical to the most conservative.

Notably, all three models displayed high predictive capability, as demonstrated by the similarities between R^2^ and PRESS R^2^, SEE, and PRESS SEE (Table 4). Beyond an extensive explanation of the variability in the DXA-derived FM% (81–85%; Figure 1), all three models proved valid by estimating a similar FM% to the reference method (*p* < 0.05) with a moderate strength of agreement (CCC = 0.90–0.92; Figure 1). The group-level accuracy was further supported by the absence of a fixed bias (~0%), while individual-level accuracy was supported by the narrow 95% LoA between the reference and the predicted FM% (fixed bias ± LoA: −2.7 to 2.8%) and by no proportional bias as indicated by the trend lines (see Figure 2). However, the Σ4SKF equation (B) offered superior accuracy to the equation resorting to the Σ3SKF (A), while the equation using the Σ8SKF (C) added no statistical or practical benefit to the Σ4SKF equation. Hence, we encourage the application of the following equation for FM% estimation:

Bettery^®^ Equation = −0.620 + 0.159 ∗ Σ4SKF (mm) + 0.120 ∗ waist girth (cm)

Using only the Σ4SKF and waist girth, our novel Bettery^®^ equation explains a larger proportion of the variability in the DXA-derived FM% than the widely used equations that were examined in our sample (Figure 1 vs. Table 2), and have been developed for male football players (85% vs. 75–76%) [5,19,28] and rugby union athletes (85% vs. 60%) [18] using the DXA as the criterion method. Moreover, all anthropometric equations cross-validated in this study have shown a greater fixed bias (all > ±0.2%) and a wider 95% LoA (all > ±2.5%) than the proposed equation (Figure 2 vs. Table 2).

Despite the positive results obtained in the present investigation, some limitations must be acknowledged. Our cross-sectional design prevents us from understanding the reliability of the novel Bettery^®^ equation. Hence, future longitudinal and interventional studies are warranted to assess its precision in tracking the FM% changes over time. Furthermore, ethnicity was not considered in this study due to the low number of players that were not Caucasian. Nonetheless, one study has previously compared Melanesian and Caucasian elite futsal players, and found no body composition differences between the groups [37], supporting our decision. Additionally, the reduced number of players in our sample with a fixed position (i.e., goalkeepers, defenders, and pivots) prevented stratification accordingly. Nevertheless, most studies have shown no differences between playing positions regarding FM or performance [1,34,38] which may be attributable to the high versatility of futsal players, with each athlete managing to fill in up to three different playing positions depending on the needs of the game [2]. Therefore, future investigations should aim to acquire larger, balanced samples to explore whether differences and significant interactions would be present for these groups and justify the contemplation of the players’ competition levels, ethnicities, or playing positions in the development of futsal-specific FM% prediction equations. Moreover, research focused on female futsal players is also important and warrants further investigation.

Furthermore, the DXA is not the most accurate existing method to assess body composition [6], though its utilisation as the criterion method in this study may also be seen as a strength. While multi-compartment models, such as the 4-compartment model, are currently viewed as true criterion methods for FM% estimation at the molecular level [39], notable logistical constraints prevent their use in most athletic settings. Therefore, by using the DXA as the criterion standard we have been consistent with more recent studies where the DXA was also used to develop FM% prediction equations [5,18,19], and with mounting evidence that encourages its usage to derive reference body-composition data [9,40,41,42].

By way of the present study, we have unlocked the possibility to use SA to estimate sport-specific FM% in adult professional and semi-professional futsal players when laboratory-based alternatives (i.e., DXA) are not available. Among other strengths of the study, the standardisation of the anthropometrist, the equipment, and the procedure (ISAK standards) resulted in a low TEM, which provides some assurance of the precision of the data. Finally, the equation has been cross-validated in a comprehensive sample of professional and semi-professional players. Therefore, the Bettery^®^ anthropometric equation for FM% estimation may be used both at the team and individual level of adult male futsal players at different competition levels.

## 5. Conclusions

The newly developed and validated Bettery^®^ prediction equation is the first to provide practitioners with an accurate and field-friendly tool to estimate FM% in professional and semi-professional male futsal players, and consequently inform training and nutrition strategies to improve body composition and futsal performance.

## Figures and Tables

**Table 1:** The participants’ anthropometric and body composition characteristics (*n* = 78).

Variables	Total Sample	Range
Anthropometry:		
Age	23 [20–30]	18–37
Body mass (kg)	72.8 ± 1.0	55.7–99.1
Stature (cm)	176.0 ± 0.8	164.0–192.0
BMI (kg/m^2^)	23.5 ± 0.2	19.1–29.5
Triceps (mm)	8.2 ± 0.3	3.7–17.0
Subscapular (mm)	9.8 ± 0.3	6.0–18.0
Bicipital (mm)	4.0 ± 0.2	2.3–10.0
Iliac crest (mm)	11.7 ± 0.6	4.0–27.5
Supraspinale (mm)	9.1 ± 0.5	3.8–23.0
Abdominal (mm)	13.1 ± 0.7	5.9–29.0
Front thigh (mm)	11.3 ± 0.5	4.8–25.0
Medial calf (mm)	5.8 ± 0.3	2.5–15.0
Sum of 3SKF (mm)	32.6 ± 1.4	15.7–68.0
Sum of 4SKF (mm)	44.3 ± 1.9	22.5–95.5
Sum of 8SKF (mm)	73.0 ± 2.9	40.1–147.0
Arm-girth relaxed (cm)	29.9 ± 0.2	25.0–35.0
Arm-girth flexed and tensed (cm)	32.5 ± 0.2	28.5–37.1
Waist girth (cm)	78.2 ± 0.5	67.4–92.3
Gluteal girth (cm)	95.7 ± 0.5	84.5–109.3
Calf girth (cm)	37.1 ± 0.3	32.1–44.4
DXA		
Bone Mineral Content (kg)	3.2 ± 0.1	2.4–4.5
Fat-Free Mass (kg)	60.0 ± 0.7	45.6–73.8
Lean Soft Tissue (kg)	56.8 ± 0.7	43.2–70.1
Fat Mass (kg)	11.4 ± 0.4	6.3–25.0
Fat Mass (%)	15.8 ± 0.4	10.5–25.6
Visceral Adipose Tissue (cm^2^)	54.5 ± 1.6	29.7–105.8

Abbreviations: 3SKF, three skinfolds (triceps, abdominal, and front thigh); 4SKF, four skinfolds (triceps, iliac crest, abdominal, and front thigh); 8SKF, eight skinfolds (triceps, subscapular, biceps, iliac crest, supraspinale, abdominal, front thigh, and medial calf); BMI, body mass index; DXA, dual-energy X-ray absorptiometry. Values are mean ± standard deviation or median (25th–75th percentiles) and range (minimum–maximum).

**Table 2:** Cross-validation of existing anthropometric equations for fat mass percentage prediction.

Author (Year)	Sample (Country)	FM%	Regression Analysis		CCC Analysis			Agreement Analysis		
		Mean ± SD	R^2^	SEE (%)	CCC	ρ	C_b_	Bias	95% LoA	Trend
Present study	78 M, high-level futsal players (PT)	15.8 ± 3.2	-	-	-	-	-	-	-	-
Athlete-specific equations										
Suarez-Arrones (2018) [19]	18 M, international elite football players (IT)	16.8 ± 3.5 *	0.75	1.61	0.83	0.8672	0.9516	1.027	−2.407; 4.461	*r* = 0.164 (*p* = 0.153)
Zemski, Caucasian (2018) [18]	26 M, elite rugby union players (AUS)	12.6 ± 2.7 *	0.60	2.05	0.48	0.7754	0.6244	−3.161	−7.164; 0.843	*r* = −0.278 (*p* = 0.014)
Reilly (2009) [5]	45 M, professional football players (UK)	10.9 ± 2.1 *	0.76	1.59	0.30	0.8718	0.3444	−4.930	−8.311; −1.549	*r* = −0.665 (*p* < 0.001)
Evans, 3SKF (2005) [27]	78 M, collegiate athletes (US)	10.2 ± 3.2 *	0.70	1.78	0.33	0.8349	0.3980	−5.560	−9.186; −1.935	*r* = −0.003 (*p* = 0.980)
Stewart (2000) [28]	82 M, local and international-level athletes (UK)	10.0 ± 4.5 *	0.75	1.63	0.39	0.8635	0.4465	−5.835	−10.520; −1.149	*r* = 0.569 (*p* < 0.001)
Withers (1987) ^1^ [29]	207 M, elite athletes from 18 sports (AUS)	10.8 ± 3.8 *	0.72	1.70	0.42	0.8508	0.4882	−4.991	−8.888; −1.094	*r* = 0.294 (*p* = 0.009)
Generalised equations										
Lohman (1981) ^1^ [30]	149 M, adults from a combination of studies (US)	7.5 ± 1.1 *	0.63	1.96	0.07	0.7964	0.0877	−8.249	−13.030; −3.467	*r* = −0.907 (*p* < 0.001)
Durnin & W. (1974) ^1^ [31]	92 M, 20–29 years old, various BMI (UK)	13.6 ± 3.6 *	0.71	1.74	0.69	0.8447	0.8219	−2.201	−6.031; 1.629	*r* = 0.224 (*p* = 0.049)
Durnin & R. (1967) ^1^ [32]	60 M, young adults (UK)	14.5 ± 3.7 *	0.71	1.74	0.78	0.8447	0.9265	−1.287	−5.131; 2.557	*r* = 0.231 (*p* = 0.042)

Abbreviations: ρ, precision; 3SKF, 3 skinfolds (triceps, abdominal and front thigh); BMI, body mass index; C_b_, accuracy; CCC, concordance correlation coefficient; FM%, fat mass percentage; LoA, limits of agreement; R^2^, coefficient of determination; SEE, standard error of the estimate. ^1^ Results from body density prediction equations were converted to FM% using Siri’s formula [20]. * Significant difference in fat mass percentage between the reference method (dual-energy X-ray absorptiometry) and the prediction equation (all *p* < 0.001).

**Table 3:** Bivariate Pearson correlation analysis between skinfolds and fat mass percentage measured by dual-energy X-ray absorptiometry (*n* = 78).

Skinfolds	DXA-Derived FM%	
	*r*	*p*-Value (2-Tailed)
Triceps (mm)	0.729	<0.001
Subscapular (mm)	0.655	<0.001
Bicipital (mm)	0.600	<0.001
Iliac crest (mm)	0.847	<0.001
Supraspinale (mm)	0.729	<0.001
Abdominal (mm)	0.895	<0.001
Front thigh (mm)	0.708	<0.001
Medial calf (mm)	0.630	<0.001

Abbreviations: *r*, Pearson correlation coefficient; DXA, dual-energy X-ray absorptiometry; FM%, fat mass percentage.

**Table 4:** Proposed fat mass percentage prediction equations for high-level male futsal players and PRESS cross-validation results (*n* = 78).

Prediction Models	Unstandardised *β*	*p*-Value	R^2^	SEE	PRESS R^2^	PRESS SEE
Σ3SKF Equation						
(Intercept)	−5.007	0.157	0.81	1.46	0.81	1.74
Σ3SKF	0.195	<0.001				
Waist girth	0.185	<0.001				
Σ4SKF Equation ^1^						
(Intercept)	−0.620	0.851	0.85	1.32	0.84	1.62
Σ4SKF	0.159	<0.001				
Waist girth	0.120	0.011				
Σ8SKF Equation						
(Intercept)	−4.486	0.225	0.80	1.51	0.80	1.80
Σ8SKF	0.092	<0.001				
Waist girth	0.174	0.001				

Abbreviations: *β*, regression coefficient; Σ3SKF, sum of 3 skinfolds (triceps, abdominal, and front thigh); Σ4SKF, sum of 4 skinfolds (triceps, iliac crest, abdominal, and front thigh); Σ8SKF, sum of 8 skinfolds (triceps, subscapular, biceps, iliac crest, supraspinale, abdominal, front thigh, and medial calf); PRESS, prediction residual error sum of squares; R^2^, coefficient of determination; SEE, standard error of the estimate. ^1^ Higher R^2^ and lower SEE denote better model accuracy.

## Data Availability

Data generated and analysed during the current study are available from the corresponding author upon reasonable request, pending approval from Bettery S.A. Requests to access the datasets should be directed to filipe.teixeira@betterylife.com.

## Supplementary Materials

The following supporting information can be downloaded at: https://www.mdpi.com/article/10.3390/nu14214514/s1, Table S1: Cross-validated anthropometric equations to estimate fat mass percentage in male futsal players.; Table S2: Development of fat mass percentage prediction models from demographic and anthropometric variables.; Table S3: PRESS cross-validation of the developed models for fat mass percentage prediction.
