# Supplementary material for: Development and Validation of an Anthropometric Equation to Predict Fat Mass Percentage in Professional and Semi-Professional Male Futsal Players
Source: Nutrients. 2022 Oct 27;14(21):4514. doi: 10.3390/nu14214514 (PMC9655567; doi:10.3390/nu14214514)
Supplement: Supplementary file 1 [file nutrients-14-04514-s001.zip › nutrients-1957386-supplementary.pdf]

**Table S1:** Cross-validated anthropometric equations to estimate fat mass percentage in male futsal players.

| Author (year) | Anthropometric Equation |
|---|---|
| **Athlete-specific equations** | |
| Suarez-Arrones (2018) | FM (%) = 8.047 + (0.616 * IC) + (0.189 * T) |
| Zemski, Caucasian (2018) | FM (%) = BF% = 5.896 + (0.265 * T) + (0.251 * Sp) + (0.394 * Ca) |
| Reilly (2009) | FM (%) = 5.174 + (0.124 * Th) + (0.147 * Ab) + (0.196 * T) + (0.13 * Ca) |
| Evans, 3SKF (2005) | FM (%) = 8.997 + (0.24658 * Σ[Ab, Th, T]) − (6.343 * Sex) − (1.998 * Race) |
| Stewart (2000) | FM (kg) = (331.5 * Ab) + (356.2 * Th) + (111.9 * BM) − 9108 |
| Withers (1987) | BD = 1.0988 − (0.0004 * Σ[T + Sb + B + Sp + Ab + Th + Ca]) |
| **Generalised equations** | |
| Lohman (1981) | BD = 1.0982 − (0.000815 * Σ[T, Sb, Ab]) + (0.0000084 * $Σ[T, Sb, Ab]^2$) |
| Durnin & W., 20-29 yrs (1974) | BD = 1.1631 − (0.0632 * log [ΣB, T, Sb, IC]) |
| Durnin & R. (1967) | BD = 1.161 − (0.0632 * log [ΣB, T, Sb, IC]) |

Abbreviations: 3SKF, 3 skinfolds (triceps, abdominal and front thigh); Ab, abdominal skinfold; B, biceps skinfold; BD, body density; Ca, calf skinfold; FM, fat mass; IC, iliac crest skinfold; Sb, subscapular skinfold; Sp, supraspinal skinfold; T, triceps skinfold; Th, front thigh skinfold; yrs, years.

All skinfolds are in millimetres. Age is in years. Race: black = 1, white = 0. Sex: female = 0, male = 1.

**Table S2:** Development of fat mass percentage prediction models from demographic and anthropometric variables ($n$ = 78).

| Prediction Models | Unstandardized $\beta$ | $P$-value |
|---|---|---|
| **Model 1** [1)] ($R^2$ = 0.77; SEE = 1.59%) | | |
| (Intercept) | 8.147 | < 0.001 |
| Σ3SKF | 0.236 | < 0.001 |
| **Model 2** [2)] ($R^2$ = 0.81; SEE = 1.46%)* | | |
| (Intercept) | -5.007 | 0.157 |
| Σ3SKF | 0.195 | < 0.001 |
| Waist girth | 0.185 | < 0.001 |
| **Model 3** [3)] ($R^2$ = 0.81; SEE = 1.47%) | | |
| (Intercept) | -4.712 | 0.186 |
| Σ3SKF | 0.200 | < 0.001 |
| Waist girth | 0.171 | 0.002 |
| Age | 0.025 | 0.424 |
| **Model 4** [4)] ($R^2$ = 0.79; SEE = 1.56%) | | |
| (Intercept) | 6.472 | < 0.001 |
| Σ3SKF | 0.241 | < 0.001 |
| Age | 0.060 | 0.060 |
| **Model 5** [1)] ($R^2$ = 0.83; SEE = 1.37%) | | |
| (Intercept) | 7.850 | < 0.001 |
| Σ4SKF | 0.180 | < 0.001 |
| **Model 6** [2)] ($R^2$ = 0.85; SEE = 1.32%)* | | |
| (Intercept) | -0.620 | 0.851 |
| Σ4SKF | 0.159 | < 0.001 |
| Waist girth | 0.120 | 0.011 |
| **Model 7** [3)] ($R^2$ = 0.85; SEE = 1.33%) | | |
| (Intercept) | -0.505 | 0.880 |
| Σ4SKF | 0.160 | < 0.001 |
| Waist girth | 0.115 | 0.022 |
| Age | 0.010 | 0.709 |
| **Model 8** [4)] ($R^2$ = 0.84; SEE = 1.37%) | | |
| (Intercept) | 7.029 | < 0.001 |

| | | |
|---|---|---|
| Σ4SKF | 0.181 | 0.000 |
| Age | 0.030 | 0.269 |
| **Model 9** 1) ($R^2$ = 0.77; SEE = 1.61%) | | |
| (Intercept) | 7.722 | < 0.001 |
| Σ8SKF | 0.111 | < 0.001 |
| **Model 10** 2) ($R^2$ = 0.80; SEE = 1.51%)* | | |
| (Intercept) | -4.486 | 0.225 |
| Σ8SKF | 0.092 | < 0.001 |
| Waist girth | 0.174 | 0.001 |
| **Model 11** 3) ($R^2$ = 0.80; SEE = 1.52%) | | |
| (Intercept) | -4.168 | 0.264 |
| Σ8SKF | 0.095 | < 0.001 |
| Waist girth | 0.160 | 0.005 |
| Age | 0.024 | 0.456 |
| **Model 12** 4) ($R^2$ = 0.78; SEE = 1.59%) | | |
| (Intercept) | 6.132 | < 0.001 |
| Σ8SKF | 0.114 | < 0.001 |
| Age | 0.056 | 0.080 |

Abbreviations: *β*, regression coefficient; Σ3SKF, sum of 3 skinfolds (triceps, abdominal, front thigh); Σ4SKF, sum of 4 skinfolds (triceps, iliac crest, abdominal, front thigh); Σ8SKF, sum of 8 skinfolds (triceps, subscapular, biceps, iliac crest, supraspinale, abdominal, front thigh, medial calf); $R^2$, coefficient of determination; SEE, standard error of the estimate.

1) Variables removed in the backward stepwise procedure: ethnicity, age, body mass, stature, arm-girth relaxed, arm-girth flexed and tensed, waist girth, gluteal girth, calf girth;

2) Variables removed in the backward stepwise procedure: ethnicity, age, body mass, stature, arm-girth relaxed, arm-girth flexed and tensed, gluteal girth, calf girth;

3) Variables removed in the backward stepwise procedure: ethnicity, body mass, stature, arm-girth relaxed, arm-girth flexed and tensed, gluteal girth, calf girth;

4) Variables removed in the backward stepwise procedure: ethnicity, body mass, stature, arm-girth relaxed, arm-girth flexed and tensed, waist girth, gluteal girth, calf girth.

* Higher $R^2$ and lower SEE denote better model accuracy

**Table S3**: PRESS cross-validation of the developed models for fat mass percentage prediction (*n* = 78).

| **Prediction Models** | **Development $R^2$** | **Development SEE** | **Validation $R^2$** | **Validation SEE** |
|---|---|---|---|---|
| **Model 1** (Σ3SKF) | 0.77 | 1.59 | 0.76 | 1.94 |
| **Model 2** (Σ3SKF, waist girth)* | 0.81 | 1.46 | 0.81 | 1.74 |
| **Model 3** (Σ3SKF, waist girth, age) | 0.81 | 1.47 | 0.81 | 1.73 |
| **Model 4** (Σ3SKF, age) | 0.79 | 1.56 | 0.77 | 1.90 |
| **Model 5** (Σ4SKF) | 0.83 | 1.37 | 0.82 | 1.72 |
| **Model 6** (Σ4SKF, waist girth)* | 0.85 | 1.32 | 0.84 | 1.62 |
| **Model 7** (Σ4SKF, waist girth, age)* | 0.85 | 1.33 | 0.84 | 1.62 |
| **Model 8** (Σ4SKF, age) | 0.84 | 1.37 | 0.82 | 1.70 |
| **Model 9** (Σ8SKF) | 0.77 | 1.61 | 0.76 | 1.98 |
| **Model 10** (Σ8SKF, waist girth)* | 0.80 | 1.51 | 0.80 | 1.80 |
| **Model 11** (Σ8SKF, waist girth, age)* | 0.80 | 1.52 | 0.80 | 1.80 |
| **Model 12** (Σ8SKF, age) | 0.78 | 1.59 | 0.77 | 1.94 |

Abbreviations: Σ3SKF, sum of 3 skinfolds (triceps, abdominal, front thigh); Σ4SKF, sum of 4 skinfolds (triceps, iliac crest, abdominal, front thigh); Σ8SKF, sum of 8 skinfolds (triceps, subscapular, biceps, iliac crest, supraspinale, abdominal, front thigh, medial calf); $R^2$, coefficient of determination; SEE, standard error of the estimate.

* Higher $R^2$ and lower SEE denote better model accuracy.
